# Human Enterovirus B: Selective Inhibition by Quinoxaline Derivatives and Bioinformatic RNA-Motif Identification as New Targets

**DOI:** 10.3390/ph15020181

**Published:** 2022-01-31

**Authors:** Silvia Madeddu, Roberta Ibba, Giuseppina Sanna, Sandra Piras, Federico Riu, Alessandra Marongiu, Annalisa Ambrosino, Paola Caria, Valentina Onnis, Gianluigi Franci, Aldo Manzin, Antonio Carta

**Affiliations:** 1 Department of Biomedical Sciences, University of Cagliari, Cittadella Universitaria di Monserrato (Cagliari), 09042 Monserrato, Italy; silvia.madeddu@unica.it (S.M.); alemarongiu91@hotmail.it (A.M.); paola.caria@unica.it (P.C.); aldomanzin@unica.it (A.M.); 2 Department of Medical, Surgical and Experimental Sciences, University of Sassari, Via Muroni, 23A, 07100 Sassari, Italy; ribba@uniss.it (R.I.); piras@uniss.it (S.P.); friu1@uniss.it (F.R.); acarta@uniss.it (A.C.); 3 Department of Biotechnology, Chemistry and Pharmacy, DoE Department of Excellence 2018–2022, University of Siena, Via Aldo Moro 2, 53100 Siena, Italy; 4 Department of Biomedical Sciences, University of Sassari, Viale S. Pietro, 43C, 07100 Sassari, Italy; 5 Department of Experimental Medicine, University of Campania “Luigi Vanvitelli”, 80138 Naples, Italy; annalisa.ambrosino@unicampania.it; 6 Department of Life and Environmental Sciences, University of Cagliari, Cittadella Universitaria di Monserrato (Cagliari), 09042 Monserrato, Italy; 7 Department of Medicine, Surgery and Dentistry “Scuola Medica Salernitana”, University of Salerno, 84081 Baronissi, Italy; gfranci@unisa.it; 8 Clinical Pathology and Microbiology Unit, San Giovanni di Dio e Ruggi D’Aragona University Hospital, 84131 Salerno, Italy

**Keywords:** enterovirus, antiviral activity, quinoxaline derivatives, coxsackievirus B, time of drug addiction, RNA-binding protein database

## Abstract

The Enterovirus genus includes many viruses that are pathogenic in humans, including Coxsackie viruses and rhinoviruses, as well as the emerging enteroviruses D68 and A71. Currently, effective antiviral agents are not available for the treatment or prevention of enterovirus infections, which remain an important threat to public health. We recently identified a series of quinoxaline derivatives that were provento be potent inhibitors of coxsackievirus B5, the most common and a very important human pathogen belonging to the enterovirus genus. We have shown how most active derivatives interfere with the earliest stages of viral replication, blocking infection. Considering the broad antiviral spectrum, a very attractive property for an antiviral drug, we aimed to investigate the antiviral activity of the most promising compounds against other Enterovirus species. Here, we investigated the susceptibility of a panel of representatives of Enterovirus genus (enterovirus A71, belonging to A species; coxsackieviruses B4 and B3; echovirus 9, belonging to B species; and enterovirus D68, belonging to D species) to quinoxaline inhibitors. We also tested cytotoxicity and selectivity indices of the selected compounds, as well as their effects on virus yield. We also investigated their potential mechanism of action by a time course assay. In addition, a bioinformatic analysis was carried out to discover potential new conserved motifs in CVB3 and CVB4 compared to the other enterovirus species that can be used as new targets.

## 1. Introduction

Enterovirus (EV) is a genus belonging to the large *Picornaviridae* family, which, at present, includes nine enterovirus species (namely, enterovirus A, B, C, D, E, F, G, H, and J) and three rhinovirus species (rhinovirus A, B, and C). Current taxonomy assignsenteroviruses infecting humans to four species: enterovirus A to enterovirus D [1]. Among these important human pathogens are poliovirus, Coxsackie virus, and rhinovirus, as well as the emerging enterovirus D68 and A71. Enteroviruses are associated with a wide spectrum of diseases ranging from the common cold, which affects millions of people every year, to severe clinical manifestations, including aseptic meningitis, encephalitis, type I diabetes, and myocarditis [2]. The most relevant agent in the EV genus is probably the poliovirus, which is also considered the prototype of the genus and responsible for acute flaccid paralysis. EV-D68 has recently emerged as an important source of severe respiratory disease worldwide. EV-A71 has been reported as the causative agent of widespread epidemics of hand–foot–mouth disease, herpangina, encephalitis, and acute flaccid paralysis [3]. Species A Coxsackievirus have been associated with flaccid paralysis due to generalized myositis, while group B Coxsackievirus (CVB) have been related to spastic paralysis, owing to important muscle injury and degeneration of neuronal tissue. CVB also infects the heart, pleura, pancreas, and liver, causing pleurodynia, myocarditis, pericarditis, and hepatitis [4,5,6,7,8,9,10], while echovirus 9 has been described as a leading cause of childhood exanthems in the summer and fall, as well as an important cause of carditis [11].

Despite many decades of intensive research, no antiviral treatment for enterovirus infections is available to date. Several antiviral drugs have been identified and proven to interfere with different steps of the viral replication process. Unfortunately, none of them progressed beyond preclinical or early clinical studies due to safety concerns or low efficacy [12,13]. Other inhibitors such as the viral 3D polymerase inhibitor (DTriP-22) or vapendavir, the viral capsid inhibitor, remain at preclinical or early clinical phases of evaluation [3]. Therefore, the only treatment for serious enteroviral infections nowadays consists of supportive care. While vaccines have succeeded in the eradication of polio, they possess no effect on non-polio enteroviral infections, which represent a continuous epidemic public health threat due to genetic diversity and the emergence of new pathogenic and resistant variants of well-known serotypes.

This highlights the importance of developing more therapeutic strategies concerning different modes of action, such as viral-entry inhibitors, which can be helpful not only for pre-exposure prophylaxis but also to protect against new variants.

In the continued search for anti-enteroviral drugs [14,15], we report herein the susceptibility of different species of human enterovirus to treatment with the most promising quinoxaline derivatives [16]. We recently showed that four compounds of a library of quinoxaline derivatives were found to be potent and selective inhibitors of an early phase of the coxsackievirus B5 cycle, with EC_50_ in the nanomolar range (300–60nM), accompanied by very low cytotoxicity [16]. In this report, we evaluated the broad-spectrum activity of these promising candidates in cell-based assays against a panel of representatives of the Enterovirus genus (enterovirus A71, belonging to A species; coxsackievirus B4 and B3; echovirus 9, belonging to B species; and Enterovirus D68, belonging to D species), and we characterized, by time-of-addition assay, their mechanisms of action. A deep RNA analysis was also performed in order to identify new antiviral targets.

## 2. Results and Discussion

### 2.1. Evaluation of Antiviral Efficacies of Quinoxaline Compounds *(**6**–**9**)* against a Representative Panel of Enterovirus Replication

**Figure 1:**
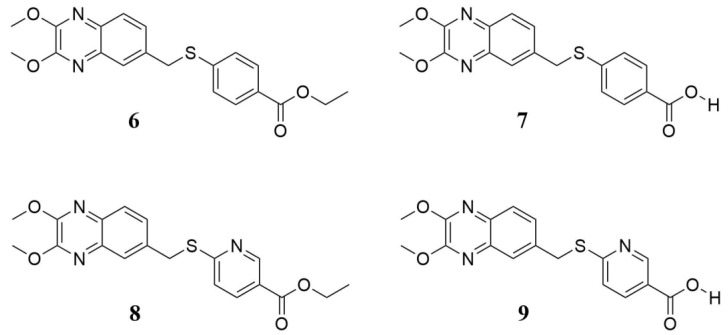
Chemical structure of quinoxaline derivatives **6**–**9** selected for deeper antiviral investigation. They are labeled as previously reported [16].

In a cell-based assay, we previously identified four interesting quinoxaline compounds (**6**–**9**), as depicted in Figure 1, that inhibited CVB5 infection in cell culture. Here, we expanded the spectrum of activity, evaluating these promising candidates against the most common and widespread enteroviruses.

As reported in Table 1, compounds **6**–**9** were tested against representatives of Enterovirus (CVB3, CVB4, EV-A71, EV-D68, and E9) in a cell-based assay. The cytotoxic effect of the compounds was also evaluated. The results showed that compounds exhibited no cytotoxicity in Vero-76 and LLC-MK2 cells, with 50% cytotoxic concentration (CC_50_) values >100 µM. Compounds 6 and 7displayed significant inhibitory activity against CVB4, with EC_50_ values of 1.7 and 1.5 µM, respectively. A comparable activity was also reported against CVB3 with an EC_50_ range of 2–3 µM. Interestingly, in vitro tests revealed a viral replication inhibition higher than 90% at a concentration of 3 µM of derivative **7** against CVB4 and 14 µM against CVB3. Compound 6 presented EC_90_ values comparable for both viruses (12 µM), CVB3 and CVB4. Furthermore, derivative **8** resulted in moderate anti-E9 activity, with an EC_50_ of 6 µM and an EC_90_ of 16 µM. However, none of the evaluated derivatives was found to be significantlyactive against the other tested enteroviruses, EV-A71 and EV-D68. Notably, derivatives **6** and **7** resulted in selectively activity against enteroviruses belonging to species B. Considering the chemical structure of the tested compounds, a structure–activity relationship (SAR) analysis can be conducted. Most certainly, the esters (**6**, **8**) and the corresponding free-acid compounds (**7**, **9**) share the same activity (or inactivity), with the sole exception of compounds **8** and **9**. When the latter were tested against E9, a one-fold loss of activity was detected compared to the parental compounds, **6** and **7**. On the other hand, the greatest difference in terms of activity was found when the thiobenzoic moiety was substituted with a thionicotinic one. A complete loss of activity against CVB3 and CVB4 was found, while even lessactivity was gained against EV-D68 and E9 EV strains. Substitution with a nitrogen atom in position 2’ promoted differences in the investigated antiviral activities, likely altering the accommodation of the aromatic ring in the binding pocket. Looking at the two thiobenzoic-based derivatives, **6** and **7**, we can point to a slight improvement when the acidic moiety is freed and not protected as an ethyl ester, while the latter can be considered a potential future pro-drug with good antiviral activity, which will not be lost after metabolic hydrolyzation of the ester group.

### 2.2. Effect of Quinoxalines on Viral Yield

**Figure 2:**
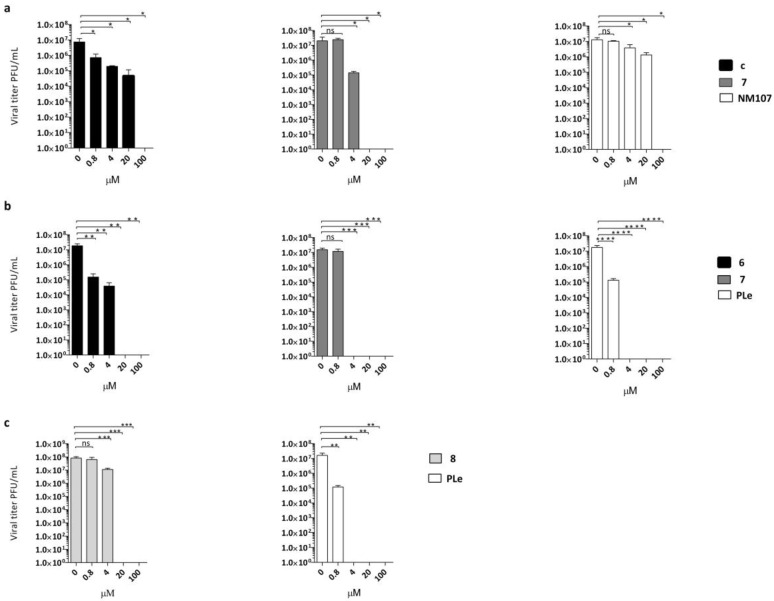
Dose-dependent reduction of (**a**) CVB3 titer in the presence of different concentrations of compound **6** (black bars) or compound **7** (dark grey bars) compared with untreated infected (no inhibitor) control and NM107 as reference (white bars) or (**b**) CVB4 titer in the presence of the same multiple concentrations of compound **6** (black bars) and compound **7** (dark grey bars), withpleconaril (PLe) used as reference control (white bars). (**c**) E9 titers in the presence of different concentrations of compound **8** (light grey bars); PLe used as reference control (white bars). The statistical significance values were defined as * *p* < 0.05, ** *p* < 0.01, and *** *p* < 0.001, **** *p* < 0.0001. Data represent mean ± standard deviation (SD) values of three independent determinations.

Antiviral activity was confirmed for three out of four of the described compounds in virus yield reduction assays (YRA) against Vero-76 and LLC-MK2 cells, as reported in Figure 2a–c. Derivatives **6**–**8** were selected as the most active out of the above-mentioned anti-EVs. Pleconaril (PLe) and NM107 (2′-C-methylcytidine), known as active against title viruses, were selected as positive reference compounds.

Notably, derivative **6** at 100 and 20 µM concentrations achieved complete suppression of CVB4 viral titers. A significant reduction was also detected at 4 µM (2.5 logs) and 0.8 µM (2 logs). The same trend of reduction in viral loads was detected for compound 6 against CVB3. The effect of derivative **7** on CVB3 and CVB4 replication was also evaluated. The production of CVB4 virus was completely inhibited at 100, 20, and 4 µM, while there was no significant inhibition of CVB4 at 0.8 µM. A dose-dependent reduction of viral titers was also observed when CVB3 was treated with the same non-cytotoxic concentrations of compound **7**. In this assay, compound **7** was found to be less active at 4 µM, with one log reduction in viral load. To prove the titer reduction of E9 when treated with derivative **8**, a YRA was carried out on LLC-MK2 cells. As showed in Figure 2c, compound **8** completely suppressed viral replication at 100 and 20 µM concentrations, whereas no reduction in viral loads at lower concentrations was detected.

### 2.3. Virucidal Activity Evaluation

To assess whether the tested compounds possessed direct virucidal activity, viral suspensions (1 × 10^5^ PFU/mL) were incubated at either 4 or 37 °C for 1 h with 20 µM of each compound. The samples were then titrated at high dilutions, at which the quinoxaline derivatives were not active. As postulated, no significant differences between the titer of viruses treated at the two different temperatures were observed (data not reported).

### 2.4. Adsorption Assay and Time Course Assay

**Figure 3:**
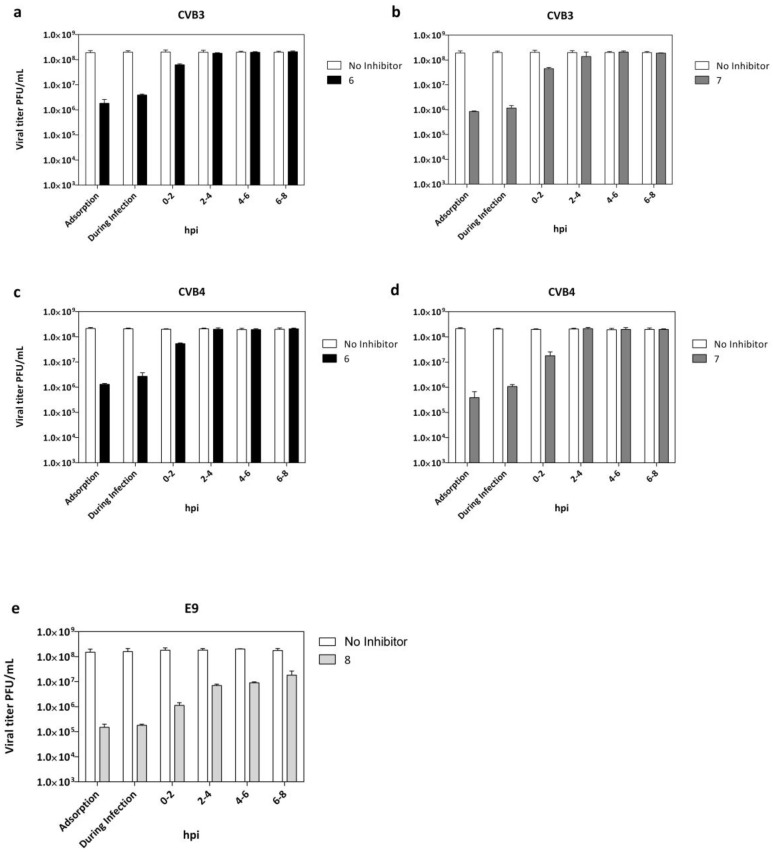
Adsorption assay and time of addition for derivatives **6** and **7** against CVB3 (**a**,**b**) and CVB4 (**c**,**d**) and derivative **8** against E9 infection (**e**). White columns: viral yield for control untreated cells; dark columns: viral yield in cells treated with compounds **6** (black), **7** (dark grey), and **8** (grey). Data are presented as mean ± SD from three independent experiments.

The step of virus replication hijacked by the tested compounds was evaluated and reported in Figure 3a–d. Vero-76 cells were incubated with CVB3 and CVB4 (m.o.i. = 0.1) and derivatives **6** or **7** for 2 h at 4 °C, using compound concentrations of 20 µM. Treatment with both compounds resulted in a detectable reduction of the virus titer in comparison to the untreated infected control. LLC-MK2 cells were incubated with E9 and derivative **8** (Figure 3e) under the above-described experimental conditions. To identify the replication stage inhibited by quinoxaline derivatives, we performed a time-of-drug addiction assay (T.O.A.) (Figure 3a–e). Experiments were carried out on CVB3-, CVB4-,and E9-infected cells (m.o.i. = 1) treated with compounds (20 µM) at different step times of infection until 12 h post-infection, followed by virus titer determination by plaque assay.

The data indicated that the maximum reduction of CVB3, CVB4, and E9 viral titers (Figure 3a–e) were observed when each tested derivative was administered at the same time of infection.

Interestingly, infectious CVB3, CVB4, and E9 particles released into media from infected cells were still reduced, even when quinoxalines were added at 0–2 h post-infection (p.i.). No significant titer reduction was detected when treatment was dispensed beyond 2 h post-infection. Importantly, targeting the virus during the early phase of infection means that significant benefit can be gained rapidly, not only for pre-exposure prophylaxis but before the progression of infection. Candidates for entry inhibition can act in a therapeutic window of opportunity before the progression of infection and the possible development of potentially fatal complications.

### 2.5. Alignment of VP1 Protein Sequences to Clarify Detected Activities amongTested EVs

**Figure 4:**
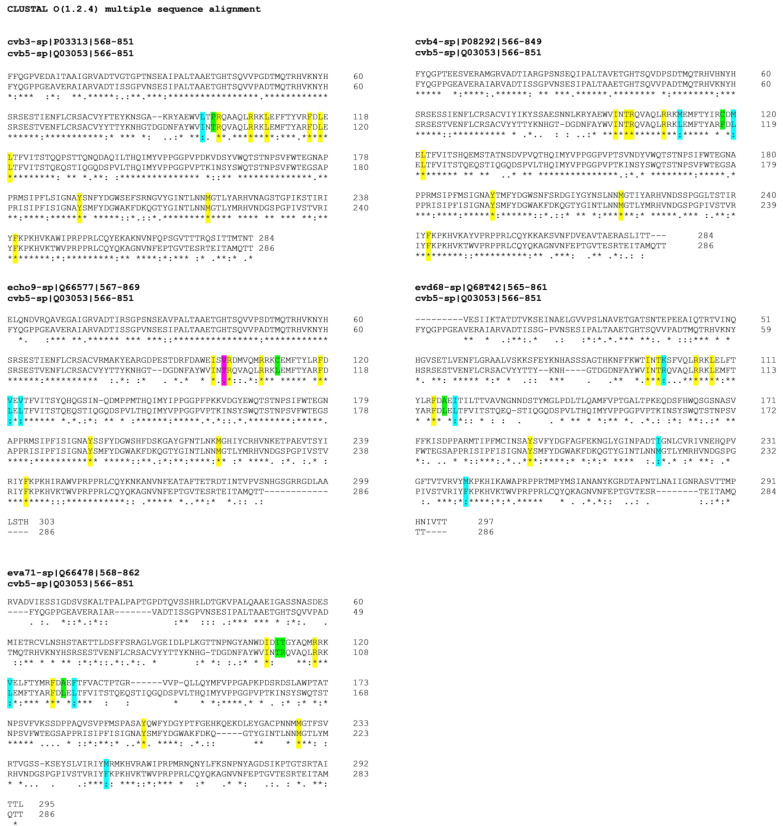
VP1 sequence of analyzed EVs, aligned with reference CVB5-VP1. Key amino acids for target-molecule binding arehighlighted with different colors for amino-acid affinities. * (asterisk) and yellow indicate positions with a single, fully conserved residue; (colon) and cyan point out conservation between groups of similar properties; (period) and magenta indicate conservation between groups of weakly similar properties; no symbol and green stand for different residues with no similar properties.

The selected quinoxaline derivatives, **6**–**9,** were reported to exert their antiviral activity by interaction with viral capsid protein VP1 [16]. Here, the protein sequences of the target VP1 among selected strains were aligned to highlight similarities and differences that could clarify the different affinities between compounds and substrates and therefore different antiviral activities. The different affinities with the target are exhibited by the variable antiviral potency of compounds **6**–**9** when tested against different EVs but also when evaluated in terms of mechanism of action. In silico molecular simulations underlined the multiple polar and nonpolar interactions established by the compounds with viral capsid protein VP1 [16]. The binding of compound **6** to the target protein is supported by several interactions with the following key amino acids of the binding pocket: I94, T96, R97, R103, L106, F114, L116, L118, Y191, M215, and F239 [16]. The protein sequences, acquired from UniProt database (http://www.uniprot.org, last access: 9 October 2020), were aligned by using CLUSTAL OMEGA online service (EMBL-EBI, European Molecular Biology Laboratory–European Bioinformatics Institute, Hinxton, Cambridgeshire, UK). Results are reported in Figure 4, and the first evidence concernsCVB3, CVB4, and CVB5, which showed the highest percentage of VP1 sequence conservation among the tested EVs, whereas E9-VP1 had a generally high homology with CVB5-VP1, with 4 residue mutations in the binding key amino acids, two of which mutated into deeply different amino acids. EV-D68, on the other hand, presented a hugely different protein sequence in general terms, but the binding key amino acids were mostly conserved, and a few residues mutated into amino acids with similar properties. EV-A71-VP1 was the least conserved among the analyzed EVs, explaining the antiviral results; none of the tested quinoxalines were able to inhibit EV-A71 replication.

### 2.6. Motif Discoverinyvia MEME Suite Tools

**Figure 5:**
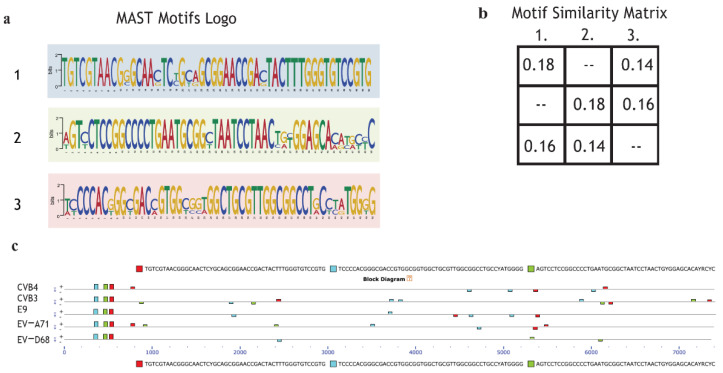
Motif de novo sequences: Motif logo of repetitive elements present in the genome of CVB4, CVB3, E9, EV-A71, and EV-D68. (**a**); Motif similarity matrix computes the pairwise correlations between each pair of motifs. The correlation between two motifs is the maximum sum of Pearson’s correlation coefficients for aligned columns divided by the width of the shorter motif. The maximum is found by trying all alignments of the two motifs (**b**). (**c**) Graphical representation of all virus genomes and the presence of motifs with the respective position across the genome.

In order to explain selective inhibition by quinoxaline derivatives, the first step of the bioinformatic analysis aimed to discover potential new motifs shared only by CVB3 and CVB4 compared to the other enterovirus species. The scan revealed that CVB4, CVB3, EV-A71, EV-D68, and E9 shared three motifs not found in the MEME database. The MAST motif logo demonstrates that the new sequences are highly conserved. Almost all the nucleotides from alignments were found in over 95% of EV genomes (Figure 5a). The second step was intended to evaluate motif coverage. The alignment showed that in all the viruses, the revealed motifs were placed in a precise structure at the 5’ end (Figure 5c). This particular scheme in a structural region suggests that the discovered sequences may have an important role in a vital process, such as the synthesis of structural proteins (capsid proteins VP1, VP2, VP3). Peculiarly, the same elements also appear in a different arrangement along the five genomes but in a non-repetitive schema and as paired and non-paired motifs (Figure 5c). The analysis did not show any particular features possessed by only enterovirus B. The significance of these preliminary findings is still unclear, but it could be interesting to evaluate the effects of a mutation or deletion to understand whether the mechanism could be involved in the different antiviral responses of CVB3, CVB4, EV-A71, EV-D68, and E9 to the administered drugs.

### 2.7. Enterovirus RNA Scanning via RNA-Binding Protein DataBase (RBPDB)

Aiming to develop further broad-spectrum antivirals, we wantto focus our future research on RNA in order to identify new targets for thedevelopment ofnew antivirals [17]. For this purpose, we performed an RNA-binding motif analysis via RNA-Binding Protein DataBase (RBPDB) [18] scan. We wanted to find any RNA-binding motifs that CVB3, CVB4 could have in common if compared to E9, EV-D68, and EV-A71. RBPDB is a database of RNA-binding specificities (http://rbpdb.ccbr.utoronto.ca/, last access: 4 April 2020) [18]. With this bioinformatic analysis, we obtained the full list of predicted motifs in all the input sequences. The first scan returned 4225 binding sites; 463 had a 100% similarity. The list was then represented in a Venn diagram. InteractiVenn offers a clean interface to build a diagram.

**Figure 6:**
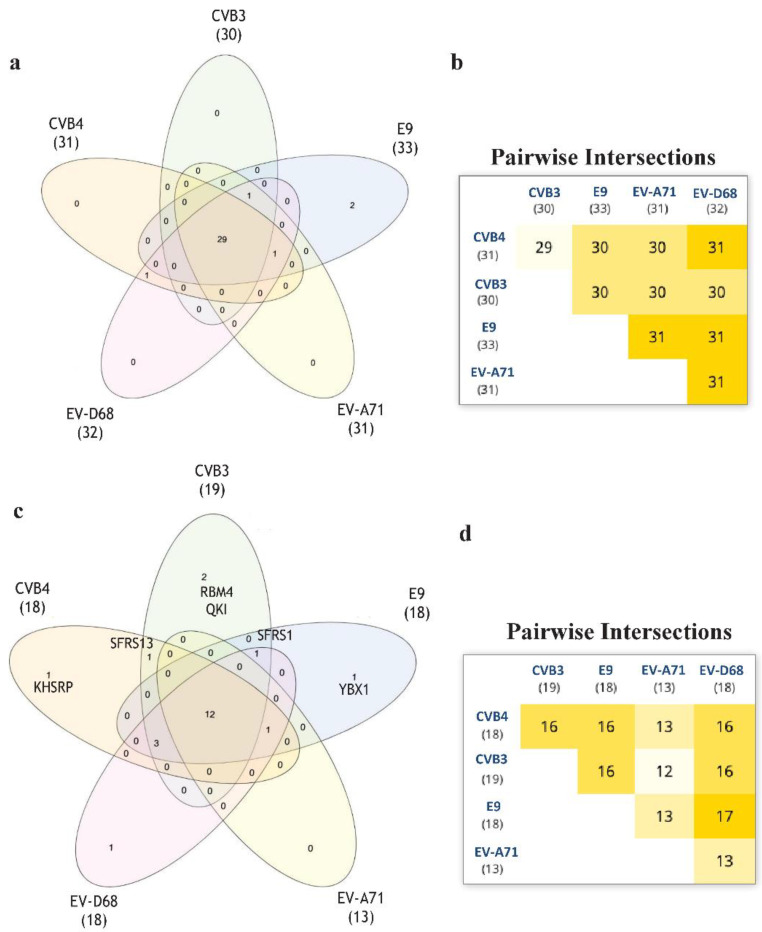
The RNA-binding motif analyzed via RBPDB approach. (**a**): Venn diagram representation for all the motifs present in the input sequences with similarity ≥80%. (**b**): Related pairwise intersection. (**c**,**d**): Venn diagram visualization and pairwise intersection of filtered motifs from the same analysis of (**a**) and (**b**), setting thefilter to obtain only motifs with 100% sequence similarity with the database used.

**Figure 7:**
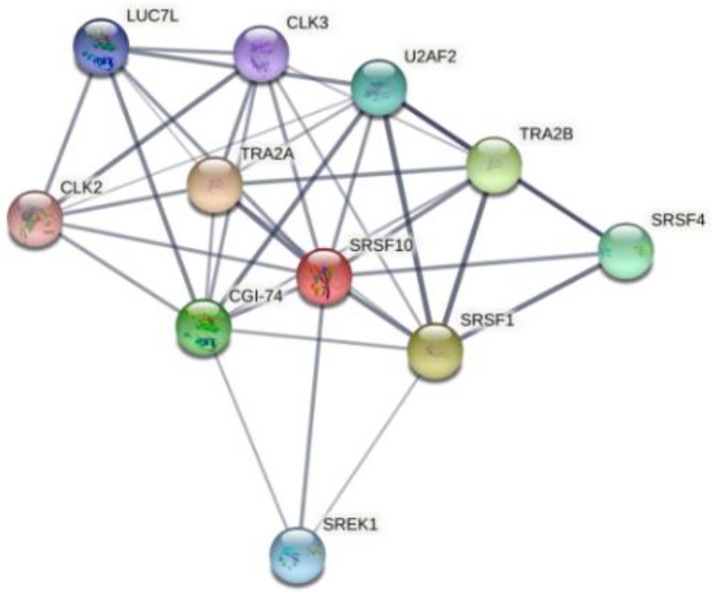
Human SFRS10 interactome network generated using STRING 11.5 database. It contains 11 nodes (proteins), with 38 edges (interactions) between them. The network shows serine/arginine-rich splicing factor, kinases, and transformer proteins.

InteractiVenn extends the ability to analyze combinations of sets of elements in part or in total, affording additional observations of the interactions between joined sets. The results are shown in Figure 6. Pairwise intersection analysis showed that the five enteroviruses share 29–31 binding motifs with a relative score greater than 80% (Figure 6a,b). A more accurate scan (default thresholds of 100%) revealed that CVB3, CVB4, E9, EV-D68, and EV-A71 have 12 sequences in common (Figure 6c,d). Interestingly, the SFRS13 sequence (a.k.a. FUSIP2, SFRS13A, TASR, SRSF10) is shared by only CVB3 and CVB4. According to the UniProt database, SFRS13 is a typical human sequence encoding for the serine/arginine-rich splicing factor 10, which is involved in mRNA processing [19,20]. In detail, when dephosphorylated (after heat shock, DNA damage, and during mitosis), it represses pre-mRNA splicing and allows exon skipping during alternative splicing. SRFS10 protein interacts with many SR, hnRNP, and kinase proteins (Figure 7) involved in mRNA splicing, processing, and stability. In particular, TRA2β, TRA2α, and U2AF2 are involved in pre-mRNA splicing; YTHDC1 and SRSF4 are alternative splicing regulators; CLK3 phosphorylates SRSF1. Thus, a change in those interactions could lead to different splicing and different transcription.

In order to understand the potential similarities in terms of motif presence, the five EVs were analyzed via the MEME suite tool. The results highlighted that the input sequences share three novel motifs found both in structural and non-structural regions. Even though the motifs are located in the same order and at the same positions at the 5’ end, it is not an unexpected result. Indeed, it is known that enteroviruses preserve a very complex and conserved 5’ UTR, which is involved in transcription, recruitment of ribosome for translation, and genome replication [21]. What is remarkable is the presence of the same sequences across genomes with different coverage and without any apparent order. It could be interesting to investigate the role of those sequences and whether a mutation or deletion could affect viral replication or pathogenicity.

Moreover, the comparison of the genomes on the RBPDP revealed that only the two enteroviruses susceptible to compounds **6** and **7**, CVB3 and CVB4, share the motif for SRSF13, which is a member of the serine/arginine-rich splicing factors involved in splicing events. In general, RNA-binding proteins play a crucial role in several important processes, such as splicing events, polyadenylation, and translation. Recent studies proved that the inhibition of SFRS13 phosphorylation causes a reduction in HBV and HIV-1 RNA levels [22,23].In addition, SFRS13 dephosphorylation increases the interaction with hTRA2β, which is an important check in HIV-1 mRNA splicing events [24,25]. In the same way, the inhibition of the kinase CLK can alter splicing events and inhibit viral replication [26]. Based on these observations, the motif for SFRS13 could be a potential new target that could increase the antiviral effect of derivatives **6** and **7**.

## 3. Materials and Methods

### 3.1. Cells and Viruses

Cell lines were purchased from American Type Culture Collection (ATCC). The absence of mycoplasma contamination was checked periodically by the Hoechst staining method. Cells supporting the multiplication of viruses were: Vero-76 [ATCC CRL 1587, *Cercopithecus aethiops*], HeLa [ATCC CCL-2 *Macaca mulatta*], and LLC-MK2 [ATCC CCL-7]. Viruses obtained from the American Type Culture Collection (ATCC) were human enterovirus B [coxsackie type B4 (CVB4), strain J.V.B. (ATCC VR-184); coxsackie type B3 (CVB3), strain Nancy (ATCC VR-30)]; human enterovirus B [echovirus 9], strain Vispo (ATCC VR-1051)l human enterovirus A71, strain BrCr (ATCC VR-1775); and human enterovirus D 68, strain Fermon (ATCC VR-1826).

### 3.2. Cytotoxicity Assays

HeLa cells were seeded in 96-well plates at a density of 5 × 10^5^ cells/mL, in minimum essential medium with Earle’s salts (MEM-E), L-glutamine, 1 mM sodium pyruvate, and 25 mg/L kanamycin, supplemented with 10% fetal bovine serum (FBS). Vero-76 and LLC-MK2 cells were seeded in 96-well plates at a density of 5 × 10^5^ cells/mL in Dulbecco’s modified eagle medium (D-MEM) with L-glutamine and 25 mg/L kanamycin, complemented with 10% FBS. Cell cultures were then incubated in a humidified, 5% CO_2_ atmosphere at 37 °C, with or without serial dilutions of analyzed compounds. The medium used for the cytotoxic assay, as well as for antiviral assays, included 1% of the proper serum. Cell viability was determined after 72–120h at 37 °C by MTT method [27].

### 3.3. Antiviral Assays

The activity of compounds against EV-A71 and D68 was based on inhibition of virus-induced cytopathogenicity in HeLa and Vero-76 cells, respectively, acutely infected at a multiplicity of infection (m.o.i.) of 0.01. After a 72 or 120 h incubation at 37 °C, cell viability was verified by the MTT method, as previously described [28]. The activity of compounds against CVB3, CVB4, CVB5, and E9 was determined in infected cell monolayers by plaque-reduction assays, as previously reported [29]. Briefly, the monolayer of Vero-76 cells or LLC-MK2 (E9) was grown overnight on a 24-well plate. The cells were then infected for 2 h with 250 μL of proper virus dilutions to obtain 50–100 PFU/well. Following removal of the unadsorbed virus, 500 μL of the medium (D-MEM with L-glutamine, 4500 mg/L D-glucose, and 1% inactivated FBS) containing 0.75% methyl-cellulose, with serial dilutions of quinoxalines, was added. The overlay was also added to untreated wells (non-infection controls). Cultures were incubated at 37 °C for 3 (CVB3, CVB4, CVB5) or 5 days (E9) and then fixed with PBS containing 50% ethanol and 0.8% crystal violet, washed and air-dried. Plaques were then counted.

### 3.4. Yield-Reduction Assay

Vero-76 and LL-MKC cells were inoculated with CVB3, CVB4, or E9 at an m.o.i. of 0.1 in maintenance medium and compounds at non-cytotoxic concentrations. After the adsorption period (2 h) at 37 °C and 5% CO_2_, the inoculum was removed and replaced with fresh medium containing the same concentration of compounds. After 72 h for CVB3 and CVB4 or 120 h for E9 at 37 °C and 5% CO_2_, each sample was collected and diluted with serial passages, starting from 10^−1^, up to 10^−10^. The titers of the supernatants were quantified by standard plaque assay. NM 107 (2’-C-Methyl-Cytidine) and pleconaril were employed as reference compounds.

### 3.5. Virucidal Activity Assay

Quinoxalines (20 µM) were incubated with 1 × 10^5^ PFU/mL of CVB3, CVB4, and E9 at either 4 or37 °C for 1 h. The mixture without a test sample was used as control. After incubation, samples were consecutively diluted in media, and titers were determined on Vero-76 cells (CVB3, CVB4) and LLC-MK2 (E9) at high dilutions, at which the compound was not active. Titers were quantified by plaque assay.

### 3.6. Time Course Assay

The monolayers of Vero-76 cells (24-well plates) were infected for 1 h at room temperature with CVB3, CVB4, and E9 dilutions to obtain a final m.o.i. of 1. After a period of adsorption, the monolayers were washed two times with DMEM medium with L-glutamine, 1% inactivated FBS, 1 mM sodium pyruvate, and 0.025 g/L kanamycin and incubated at 5% CO_2_ at 37 °C (time zero). Vero-76 cells (CVB3, CVB4) and LLC-MK2 cells (E9) were treated with quinoxalines (20 μM, approximately 10 times higher than IC_50_) or reference for 1 h during the infection period (at −1 to 0) and at a defined time point, 0 to 2, 2 to 4, 4 to 6, or 6 to 8 h post-infection. After each incubation phase, the monolayers were washed two times with medium or buffer and incubated with fresh medium until 12 h post-infection. Then, the monolayers were frozen at −80 °C, and the viral titers were determined by plaque assay.

### 3.7. Adsorption Assays

Vero-76 and LLC-MK2 cells grown in 24-well plates were infected with CVB3, CVB4, and E9, at an m.o.i. of 0.1, with or without test compounds, and incubated for 120 min at 4 °C. Medium containing unbound virus was then removed, and cells were washed twice with PBS and overlayed with fresh medium. Plaques were counted after 72 h of incubation at 37 °C.

### 3.8. Statistical Analysis

Cell-based assays were independently repeated at least three times. The results are reported as mean ± standard deviation (SD). The statistical significance values were defined as * *p* < 0.05, ** *p* < 0.01, *** *p* < 0.001, and **** *p* < 0.0001. Statistical significance was calculated with the statistical unpaired student’s *t*-Test, performed in GraphPad Prism (San Diego, CA, USA).

### 3.9. Linear Regression Analysis

The size of cell growth/viability and viral multiplication at each tested drug concentration was expressed as percentage of untreated controls. Concentrations resulting in 50% inhibition (CC_50_ or EC_50_) were determined by linear regression analysis.

### 3.10. VP1 Protein Alignment

Protein sequence alignment was performed by using CLUSTAL OMEGA software (https://www.ebi.ac.uk/Tools/msa/clustalo/, last access: 9 October 2020) [30]. Protein sequences were acquired from theUniProt website (https://www.uniprot.org/, last access: 9 October 2020), and it should be highlighted that the CV-B5-VP1 deposited sequence had anamino-acid numbering increase of 3 units.

### 3.11. Novel Motif Discoveryvia MEME Suite Tools

Enterovirus genomes were analyzed through the MEME suite tool (https://meme-suite.org/, last access: 4 April 2020) to highlight novel motifs and similarities between them that could explain their different antiviral responses. In addition, the occurrence and localization of the new sequences was evaluated with MAST [31]. Analyses were performed following default parameters. MAST version 5.1.1 was used (Release date: Wed Jan 29 2020).

### 3.12. Enterovirus RNA Scanning via RNA-Binding Protein DataBase

Enterovirus RNA wasscanned via RNA-Binding Protein DataBase (RBPDP) (http://rbpdb.ccbr.utoronto.ca/, last access: 5 April 2020) to highlight the binding motifs shared by input sequences [18]. Firstly, the matching lines with a relative score greater than the threshold of 80% were chosen. Then, the default threshold was increased to 100% to obtain RNA-binding motifs with higher sequence similarity. In both cases, to explore differences and similarities between the input sequences, Venn diagrams were plotted via the InteractiVenntool (http://www.interactivenn.net/, last access: 5 April 2020) [32]. The predicted protein–protein interactions wereevaluated via STRING database (https://string-db.org/, last access: 2 September 2021) [33].

## 4. Conclusions

Encephalitis, sepsis, poliomyelitis, acute heart failure, and myocarditis are typical manifestations of enterovirus infections in humans. Among the enteroviruses, coxsackievirus B results in medically important pathogens. They are known to be related to spastic paralysis, as well as human central nervous system and cardiac diseases. With no approved antiviral treatment, research on new valuable molecules able to fight these severe diseases is crucial, given their important clinical impact.

In the current study, we evaluated the broad-spectrum activity of quinoxaline derivatives in cell-based assays against a panel of representatives of Enterovirus. We identified interesting anti-enterovirus B agents with significant antiviral activities at non-cytotoxic concentrations. Derivatives **6**, **7**, and **8** exhibited the highest inhibitory activity during the entry of the virion into the host cell by interacting with the VP1 capsid protein. Parallelly, we ran a bioinformatics analysis to discover potential new motifs in CVB3 and CVB4 when compared to the other enterovirus species.

The analysis exhibited a potential antiviral target on mRNA shared by only CVB3 and CVB4. In silico prediction of RNA–protein interactions prompted us to hypothesize that changes in this site may have an additive effect at the level of mRNA splicing, thus causing a reduction in virus replication.

Although selected quinoxaline derivatives inhibited CVB3, CVB4, and E9 infections, with EC_50_ values in the low micromolar range, our findings on the potential mechanism of action call for further investigation to develop more interesting derivatives or combination treatments.

## Figures and Tables

**Table 1:** Cytotoxicity and antiviral activity of quinoxaline derivatives and reference compounds against representatives of Enterovirus (CVB4, CVB3, EV-A71, EV-D68, and E9).

Cmp	Vero76	CVB4	CVB4	CVB3	CVB3	EV-A71	HeLa	EV-D68	LLC-MK2	E9	E9
	^a^CC_50_	^d^EC_50_	^e^EC_90_	^d^EC_50_	^e^EC_90_	^f^EC_50_	^b^CC_50_	^g^EC_50_	^c^CC_50_	^d^EC_50_	^e^EC_90_
6	>100	1.7	12	2.5	13	>100	>100	>100	>100	>100	Nd
7	100	1.45	3.2	2	14	>100	95	>95	100	>100	Nd
8	>100	>100	nd	>100	nd	>100	>100	50	>100	6	16
9	100	>100	nd	100	nd	>100	>100	70	100	55	Nd
PLe	>100	2 ± 1	-	-	-	-	>100	0.4 ± 0.2	>100	0.1 ± 0.05	-
NM107	>100	-	-	29 ± 3	-	6 ± 1	-	-	-	-	-

^a–c^ Compound concentration (µM) required to reduce the viability of mock-infected Vero-76 cells, HeLa, and LLC-MK2by 50%, as determined by the MTT method after 3 days and 5 days, respectively. ^d^ Compound concentration (µM) required to reduce the plaque number of CVB4, CVB3m and E9 by 50% in Vero-76 and LLC-MK2 cells. ^e^ Compound concentration (µM) required to reduce the plaque number of CVB4, CVB3, and E9 by 90% in Vero-76 and LLC-MK2 cells. ^f^ Compound concentration (µM) required to achieve 50% protection of Vero-76 cells from EV-A71-induced cytopathogenicity, as determined by the MTT method at day 4/5 post infection (p.i.). ^g^ Compound concentration (µM) required to reduce the viability of mock-infected HeLa cellsby 50%, as determined by the MTT method after 3 days p.i. Pleconaril (PLe) and NM107 (2′-C-methylcytidine) were used as reference controls.

## Data Availability

The data presented in this study are available in article.
